# The Occurrence or Fibrillary Glomerulonephritis in Patients with Diabetes Mellitus May Not Be Coincidental: A Report of Four Cases

**DOI:** 10.1155/2013/935172

**Published:** 2013-05-20

**Authors:** Fayna González-Cabrera, Fernando Henríquez-Palop, Ana Ramírez-Puga, Raquel Santana-Estupiñán, Celia Plaza-Toledano, Gloria Antón-Pérez, Silvia Marrero-Robayna, Davinia Ramírez-Medina, Roberto Gallego-Samper, Nicanor Vega-Díaz, Rafael Camacho-Galan, José C. Rodríguez-Pérez

**Affiliations:** ^1^Nephrology Department, Dr. Negrin University Hospital, Las Palmas de Gran Canaria University, Barranco de la Ballena s/n, 35010 Las Palmas de Gran Canaria, Spain; ^2^Nephrology Department, University Hospital Materno-Insular, Spain; ^3^Pathology Department, Dr. Negrin University Hospital, Las Palmas de Gran Canaria University, Barranco de la Ballena s/n, 35010 Las Palmas de Gran Canaria, Spain

## Abstract

Although clinical presentation of fibrillary glomerulonephritis is similar to most forms of glomerulonephritis, it is usually difficult to make the diagnosis. Clinical manifestations include proteinuria, microscopic haematuria, nephrotic syndrome, and impairment of renal function. A diagnosis of fibrillary glomerulonephritis is only confirmed by renal biopsy and it must comprise electronmicroscopy-verified ultrastructural findings. We report four cases between 45–50 years old with documented type 2 diabetes mellitus (T2DM) and arterial hypertension. All patients were found to have fibrils on kidney biopsy. The differential diagnosis of fibrils in the setting of diabetes mellitus is also discussed.

## 1. Introduction

Fibrillary glomerulonephritis (FGN) is a rare entity, first described by Rosenmann and Eliakim in 1977 [1]. The clinical presentation may be similar to most forms of glomerulonephritis and usually with a difficult clinical diagnosis. Nephrotic syndrome is the most common clinical presentation. In optical microscopy, we observe different patterns: membranoproliferative, mesangial proliferative, glomerular sclerosis, and thickening of the basement membrane or capillary tufts [2]. Immunofluorescence reveals IgG deposits, predominantly IgG4, C3, kappa (*κ*) and lambda (*λ*) light chains. Definitive diagnosis requires the use of electron microscopy. An ultrastructural finding is a deposit of microfibril in the glomerular basement membrane or the glomerular capillary wall, basically in the subepithelial region and less frequently in the tubular basement membrane. The fibrillar component acquires focal accumulations of electron-dense deposits corresponding to immunoglobulins and complement. FGN belongs to a group of glomerular diseases known as fibrillary glomerulopathies. This group with the ultrastructural feature of nonbranching microfibril deposition within glomeruli includes amyloidosis, light chain disease, cryoglobulinemia, systemic lupus erythematosus, diabetic fibrillosis, immunotactoid glomerulopathy, and FGN. The differences with amyloidosis are the absence of staining with Congo red and with immunotactoid glomerulonephritis; it differs in the size of the fibrils, which are larger and of parallel arrangement [3].

## 2. Case Reports

### 2.1. Case 1

**Figure 1:**
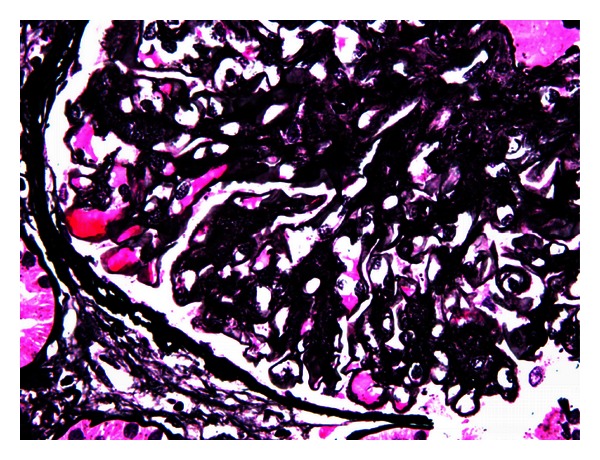
Methenamine silver—periodic acid—Schiff stain (×600): the appearance is distinctive moth eaten in the mesangial matrix and thickening capillary walls.

**Figure 2:**
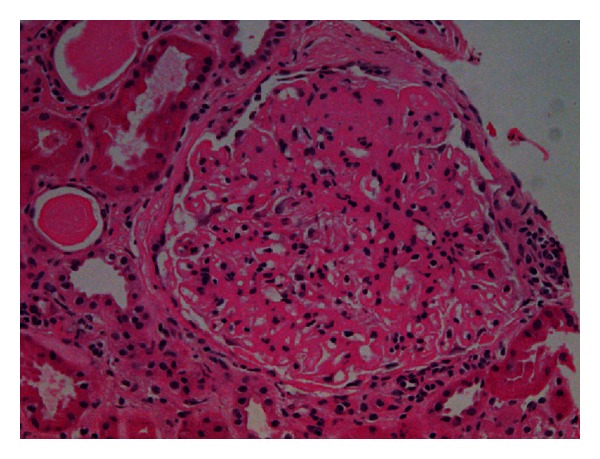
Hematoxylin-eosin stain (×400) shows accumulation of amorphous acidophilic extracellular material in mesangium and capillary walls.

**Figure 3:**
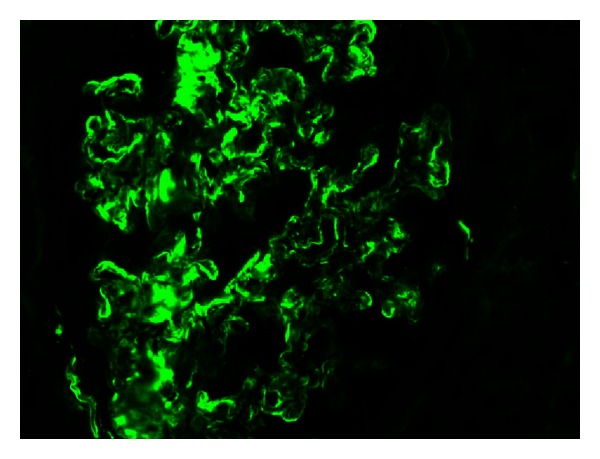
Immunofluorescence microscopy (×400) shows IgG band like capillary wall deposits.

**Figure 4:**
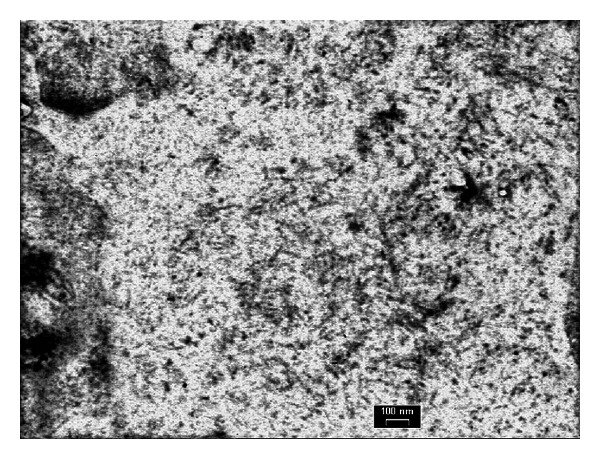
Ultrastructural study (1592 × 11 mr) exhibit straight and nonbranching fibrils ranging in diameter from 15–25 nm.

A 55-years old white male with type 2 diabetes mellitus (T2DM) since 5–7 years ago, as well as photocoagulated retinopathy, hypertension, paroxysmal atrial fibrillation with circumferential pulmonary vein ablation, gout, psoriasis, hypothyroidism due to amiodarone, and bilateral hip prosthesis. He developed nephrotic syndrome. Serum creatinine level was 1.1 mg/dL, eGFR (CKD-EPI) 62.7 mL/min/1.73 m^2^, urine protein excretion 13.52 g/d, total cholesterol 324 mg/dL, HDL-chol 35 mg/dL, LDL-chol 289 mg/dL, and albumin 24.44 g/L. Physical examination showed no edema. The kidney biopsy generated 14 glomeruli, one with global sclerosis. Glomerular basement membrane thickening of the capillary wall and increase in mesangial matrix as well as spikes in some glomeruli were shown on light microscopy suggesting a membranous nephropathy. Other images like “railroad tracks” with silver stains, low tubulointerstitial fibrosis with tubular atrophy, and chronic inflammation were also seen (Figures 1 and 2). Immunofluorescence (Figure 3) showed diffuse patchy granular and mesangial IgG, C3, and *κ* deposits. Electron microscopy showed extensive glomerular deposition of electron-dense fibrils in the mesangium and glomerular basement membrane: fibril ranging from 15 to 20 nm (Figure 4). Congo red stains were negative and a diagnosis of FGN was made. The patient was treated with dual RAS blockers, statins, omega 3 fatty acids, and depletive therapy with torasemide, amiloride, and hydrochlorothiazide. Three months later, due to unremitting nephrotic syndrome, we offered the patient to add prednisone 20 mg/d and cyclophosphamide 2 mg/kg/d for 6 months, with no response. Despite persistent nephrotic syndrome, renal function (Ccr and eGFR) did not change.

### 2.2. Case 2

A 46-year-old white woman with 12–15-year duration of T2DM and arterial hypertension developed edema and proteinuria of 4.58 g/d. Serum creatinine level was 0.93 mg/dL and eGFR (CKD-EPI) 87.4 mL/min/1.73 m^2^. Physical examination findings were unremarkable except for blood pressure of 150/90 mmHg and trace pedal edema. Laboratory findings showed normal complement, no antinuclear or double-stranded DNA antibodies, no cryoglobulins, and normal protein electrophoresis. Total cholesterol was 228 mg/dL, HDL-chol 35 mg/dL, and triglycerides 511 mg/dL. The kidney biopsy generated 18 glomeruli, 38% with global sclerosis with two fibrotic crescent formations. Light microscopy showed an increase in mesangial matrix, glomerular basement membrane thickening, fusty looking capillary walls, segmental tubular atrophy, and nonspecific chronic inflammation, as a membranous glomerulonephritis. Congo red stains were negative. Immunofluorescence showed diffuse patchy granular and mesangial *κ*, *λ*, IgG (++++), IgM (+) and complement (+), IgA, and fibrinogen were negative. Electron microscopy showed two glomeruli with extensive glomerular deposition of electron-dense fibrils in the mesangium, subepithelial, intramembranous, and subendothelial spaces. Fibrils were randomly arranged and 17 nm in thickness. A diagnosis of FGN with focal segmental glomerulosclerosis was made. Patient initially received one RAS blocker, statin, and low protein diet, and nowadays we have added a second drug for dual angiotensin II blockade (ramipril plus losartan). Her creatinine level is 1.0 mg/dL and urine protein is 2.57 g/24 h after 7 months monitoring.

### 2.3. Case 3

A 46-year-old white man presented with a diagnosis of T2DM 10 years ago with metadiabetic complication, hypertension, and obesity (BMI 34). He developed renal impairment with serum creatinine level of 1.8 mg/dL and eGFR (CKD-EPI) of 43.3 mL/min/1.73 m^2^ with a nephrotic range proteinuria (urine protein 10.39 g/d). He has a diagnosis of chronic osteomyelitis after a femur fracture with chronic antibiotic therapy. His physical examination was normal with minimal lower extremity edema. Blood pressure average was 140/90 mmHg. Urinalysis showed 3+ proteins and no microscopic hematuria. Antinuclear antibodies, double-stranded DNA antibodies, and antinuclear cytoplasmic antibodies were absent. Total cholesterol was 135 mg/dL, under treatment with statin. A kidney biopsy was performed (15 glomeruli), 3 with global sclerosis. Light microscopy showed an increase in mesangial matrix, 35% of interstitial fibrosis as a membranoproliferative glomerulonephritis. Stains were negative for Congo red. Immunofluorescence (9 glomeruli) showed global deposits that stained 3+ for IgG, 3+ for C3, *κ* and *λ*. Electron microscopy showed capillary basement membrane thickening and mesangial matrix with gross deposits, fibrillary structure, a diameter between 12 and 22 nm, and haphazardous arrangement. We saw foot process fusion of visceral epithelial cells. A diagnosis of FGN was made. The patient received angiotensin receptor blocker, chronic antibiotic therapy, and statins. Due to osteomyelitis, we have not so far introduced corticosteroid and immunosuppressive treatment.

### 2.4. Case 4

A 55-years old white male with T2DM and arterial hypertension since 5–7 years ago. He developed renal impairment with serum creatinine level of 1.55 mg/dL and eGFR (CKD-EPI) of 50 mL/min/1.73 m^2^ with nonnephrotic range proteinuria (2.66 g/d). Physical examination showed a high blood pressure and pitting edema. A blood sample analysis showed total cholesterol 194 mg/dL and triglycerides 226 mg/dL, and microhematuria was detected in the urine sample. Protein electrophoresis, immunoglobulins with other autoimmunity, and serological studies (including hepatitis B and C) were within the normal range. Kidney biopsy showed 11 glomeruli, 4 of them completely sclerotics. Light microscopy showed focal and segmental glomerulosclerosis changes, with slight fibrosis. Congo red stain was negative. Immunofluorescence showed IgG, complement, and *λ* light chains. Electron microscopy exhibits fibril deposits with 21–25 nm fibrillary structures of random arrangement. A diagnosis of FGN was made. We administered antiproteinuric treatment with dual RAS blockade with nonimmunosuppressive treatment. The renal function and proteinuria improved at the beginning of the therapy, and monitoring has shown slow progression of the kidney function five years after diagnosis. 

## 3. Discussion

FGN is a rare or an underdiagnosed entity. FGN is found in 0.5–1% of native kidney biopsies. It is more frequent in Caucasian people between 50 and 60 years old. Many cases of FGN are idiopathic, but it has been associated with malignancies (multiple myeloma, leukaemia, or solid tumours) or autoimmune or systemic diseases (idiopathic thrombocytopenic purpura, ankylosing spondylitis, Sjögren, etc.) including T2DM (Table 1) in 20% of patients and called by someone as fibrillary glomerulopathies because of nonbranching microfibril deposition [4]. The fibril deposition is generally limited to the kidney, but some patients with extrarenal deposits have been described [5, 6], suggesting that FGN is a systemic disease. Most authors believe that FGN and immunotactoid glomerulopathy are separate disorders, but it may be difficult to distinguish between both [7], resulting in the last image setting of microtubules in parallel arrays in the range of 30–40 nm.

Most patients present proteinuria from the beginning, which is usually in nephrotic range. Renal insufficiency, hematuria, and hypertension are very often as well [4] and most series described progression to end stage of renal disease in approximately half of the patients within a few years after diagnosis. 

The light microscopic findings in this condition are quite variable. Most cases reveal mesangial expansion and some mesangial cell proliferation whereas in other cases, mesangial deposition of eosinophilic material, difficult to be differentiated from that seen in amyloidosis, represents the most noticeable finding. In most cases, there is also thickening of peripheral capillary walls, which may create confusion with a membranous nephropathy. The expanded mesangial areas which are silver negative and of a moth-eaten appearance similar to that described in amyloidosis may be seen. About 15% to 20% of cases with FGN have crescents [3, 8, 9]. Immunofluorescence reveals a diffuse and coarsely granular staining for IgG, C3, *κ* and *λ* chains along peripheral capillary walls and mesangium. These findings suggest an immune mediated mechanism. Whether IgG typification is performed, IgG4 is dominant in most FGN. We need electron microscopy to confirm the diagnosis. Ultrastructural examination identifies randomly disposed 15 to 25 nm diameter fibrils along peripheral capillary walls, frequently in the mesangium and also in the interstitium.

The pathogenesis of this disorder has been a source of controversy over the years. It is believed that the fibrils occur because of polymerization of immune complexes and possibly monoclonal light chains in some cases. The fact that a particular IgG (IgG4) is dominant in most cases probably represents a relevant consideration, which may imply that this particular immunoglobulin in the proper setting can polymerize into fibrils [3, 8, 9].

Is there any association between FGN and diabetes mellitus or is it just a coincidence? 

One of the most surprising aspects of our study was that all our patients were diabetic (T2DM). Meanwhile, nondiabetic renal lesions are encountered in 34–40% [10], whereas FGN coexist in 20% of T2DM patients. This reveals a greater susceptibility of diabetic patients to immune complex injury [11]. That issue makes us wonder if there could be some type of relationship between both entities. The diabetic nephropathy is the most important cause of end stage renal disease in our field. The development of proteinuria and renal impairment in diabetic patient (almost if he has hypertension as well) usually suggests diabetic nephropathy. However, we should consider other causes of renal disease when the followup of diabetic nephropathy is unusual. Nowadays, we have more data and know how heterogeneous the clinical presentation of diabetic nephropathy is, as well as how the new research evidence generated from human diabetic kidney disease and from experimental models highlights the importance of podocytopathy in the pathogenesis of diabetic nephropathy [12]. Recently, a few studies which described FGN in T2DM patients [13, 14] have been published, but nobody talks about a possible pathogenic coincidence between both entities. It has been suggested that accelerated glycosylation of proteins in diabetics and advanced glycosylation end products are capable of cross-linking with other structural proteins as well as circulating proteins [15]. As diabetic nephropathy is quite common, and virtually any glomerular disease can coexist with it, our data might suggest to be more than simply coincidental, but this hypothesis has not been confirmed yet. Most groups assume diabetic nephropathy to be a clinical diagnosis without biopsy in human beings. 

In the last up-to-date overview of diabetic nephropathy, George Bakris stressed the importance of elucidating a relatively frequent group of nondiabetic renal disease, being superimposed in diabetic people, which may often be underestimated. He suggested that the major clinical clues suggesting the presence of nondiabetic glomerular disease are [16, 17] as follows:

- the onset of proteinuria less than five years from the documented onset of T1DM, since the latent period for overt diabetic nephropathy is usually at least 10 to 15 years. The latent period is probably similar in patients with T2DM, but the time of onset is often difficult to ascertain,
- the acute onset of renal disease. Diabetic nephropathy is a slowly progressive disorder characterized by increases in protein excretion and the serum creatinine concentration over a period of years,
- the presence of active urinary sediment, containing red cells (particularly acanthocytes) and cellular casts. However, hematuria and red cell casts can also be seen with diabetic nephropathy alone [18],
- in contrast to T1DM, lack of retinopathy in type 2 diabetes does not preclude diabetic nephropathy, which was absent in 12 of 27 patients with biopsy confirmed diabetic nephropathy in one study,
- signs and/or symptoms of another systemic disease [16],
- significant reduction in the glomerular filtration rate (>30 percent) within two to three months of the administration of ACE inhibitors or angiotensin II receptor blockers [17].

The most common nondiabetic glomerular diseases described in studies were membranous nephropathy, IgA nephropathy, postinfectious glomerulonephritis, minimal change disease, or focal glomerulosclerosis. These disorders typically have a relatively rapid onset in contrast to the slow progression over years from microalbuminuria to macroalbuminuria in diabetic nephropathy. For these reasons, in recent years, most of the university hospitals are less restrictive in indicating renal biopsy. We had described 4 cases with different presentations, treatment, and evolution in patients with T2DM and FGN. We suggest that FGN could be one of those nondiabetic diseases superimposed to diabetic nephropathy. For these reasons, we are convinced that we are able to give a better patient care by performing more renal biopsies, especially in those patients in whom we observe different clinical aspects which make diabetic nephropathy less likely.

There is currently no effective therapy for patients with FGN. There have been no controlled trials that agree on recommending an ideal therapy, and most reports choose the treatment according to light microscopy findings. Different agents have been proposed as treatment of this rare entity: corticosteroid or cytotoxic agents have been used with limited effectiveness [19]; some groups have published their successful experience using mycophenolate mofetil, rituximab concomitantly with corticosteroids, immunoglobulin, or calcineurin inhibitors [4, 20, 21]. There is controversy in relation to the use of plasmapheresis as a therapy. Schwartz et al. [22] showed no therapeutic effects of plasmapheresis in three patients with nephrotic syndrome due to immunotactoid glomerulopathy, but Pliquett et al. reported an important proteinuria reduction after initial plasmapheresis cycles [23]. Prospective multicentric studies are needed to learn more about FGN and its connection with diabetic nephropathy and to determine the optimal therapeutic regimen for this unusual disease.

## Figures and Tables

**Table 1:** Clinical, biochemical, and histologic data from patients with fibrillary glomerulonephritis.

No.	Sex	Age	SCr	Proteinuria	Haematuria	TCh	LDL	TGC	Ua	LM	SG	IF	EM (fibrils)	DM	IS
1	M	55	1.02	13.52	Yes	319	49	165	4.21	MG	1/14	IgG, C, and *κ*	15–20 nm	Yes	Yes
2	F	48	0.93	4.58	Yes	178	—	117	6.35	MG	7/18	IgG, IgM, *λ*, and C	17 nm	Yes	No
3	M	45	1.85	9.33	No	228	—	514	6.21	MP	3/15	IgG, *κ*, and *λ*	18.22 nm	Yes	No
4	M	55	1.54	2.66	sí	194	108	40	5.2	GSF	4/11	IgG, *λ*, and C	21–25 nm	Yes	No

SCr: serum creatinine at diagnosis in mg/dL; Proteinuria: urine protein at diagnosis in g/day; Haematuria: microhematuria; TCh: total cholesterol; LDL: LDL cholesterol; TGC: triglycerides; Ua: uric acid; LM: light microscopy; SG: sclerotic glomeruli; EM: electron microscopy; IF: immunofluorescence; DM: diabetes mellitus; IS: immunosuppressive treatment; MG: membranous glomerulonephritis; MP: membranoproliferative glomerulonephritis; EFG: focal and segmental glomerulosclerosis.
